# Bis(4′-chloro-2,2′:6′,2′′-terpyridine-κ^3^ *N*,*N*′,*N*′′)ruthenium(II) dichloride dihydrate

**DOI:** 10.1107/S1600536812021459

**Published:** 2012-05-16

**Authors:** Ying Wang, Rui Jiao, Xiang-Lei Qiu, Jian Wang, Wei Huang

**Affiliations:** a Department of Chemistry and Chemical Engineering, Lianyungang Teacher’s College, Lianyungang 222006, People’s Republic of China; b State Key Laboratory of Coordination Chemistry, Coordination Chemistry Institute, School of Chemistry and Chemical Engineering, Nanjing University, Nanjing 210093, People’s Republic of China

## Abstract

In the cation of the title compound, [Ru(C_15_H_10_ClN_3_)_2_]Cl_2_·2H_2_O, the metal atom exhibits a distorted octa­hedral coordination geometry provided by the N atoms of two tridentate terpyridine ligands. The ligands are approximately planar [maximum deviation = 0.156 (5) Å] and form a dihedral angle of 87.0 (3)°. In the crystal, the cations, anions and water mol­ecules are linked into a three-dimensional network by C—H⋯Cl, C—H⋯O and O—H⋯Cl hydrogen bonds.

## Related literature

For the structures of the related hydro­chloride tetra­fluoridoborate and hydro­chloride hexa­fluorido­phospho­rate derivatives, see: Huang & Qian (2007*a* ▶). For the structures of Ru^II^, Cu^II^, Zn^II^, Ni^II^, Fe^II^, Cu^II^ and Cd^II^ complexes of 4′-chloro-2,2′:6′,2′′-terpyridine, see: Beves *et al.* (2008 ▶); Huang & Qian (2007*b* ▶); Huang *et al.* (2009 ▶); You *et al.* (2008 ▶); You *et al.* (2009 ▶).
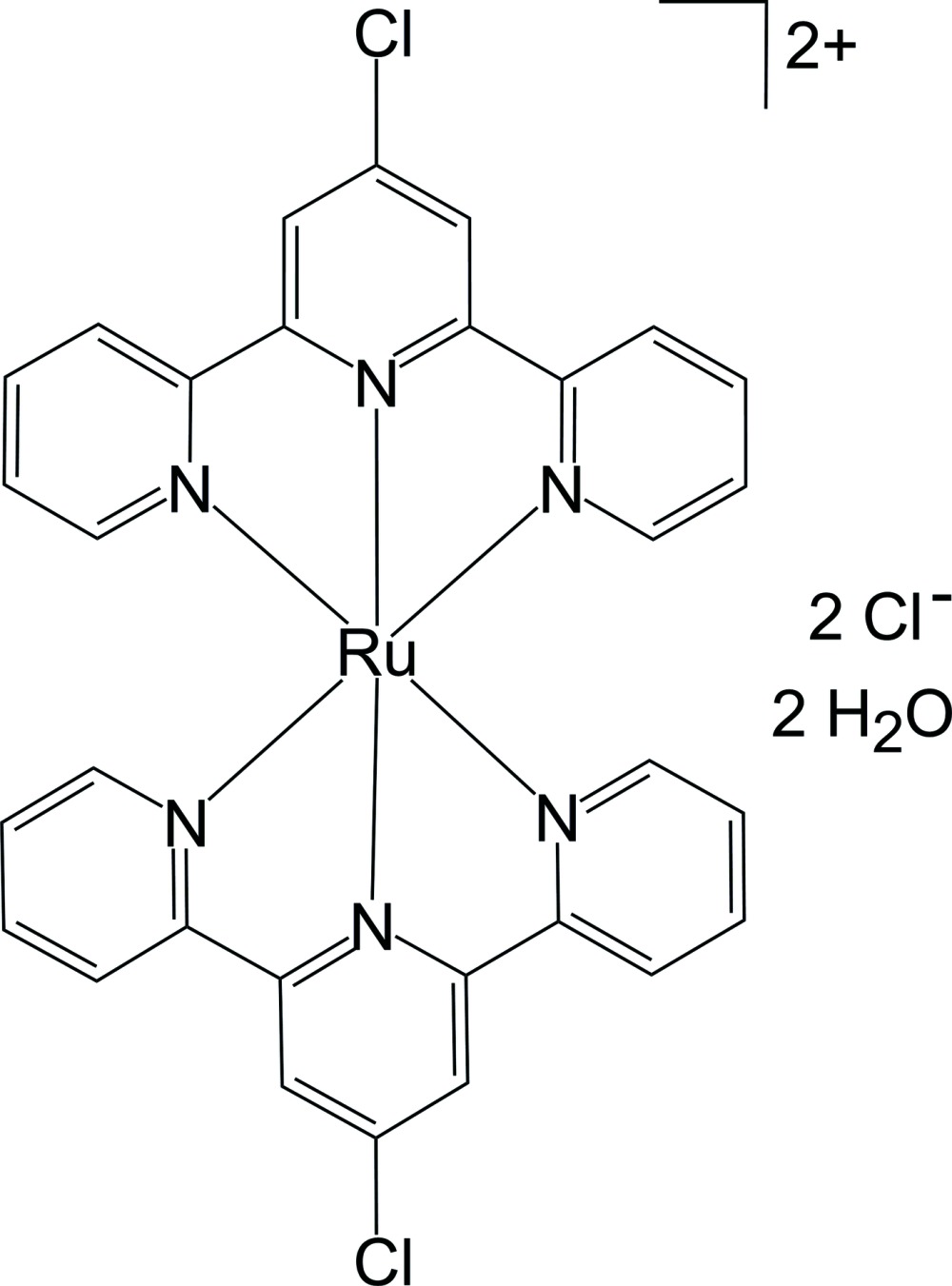


## Experimental

### 

#### Crystal data

[Ru(C_15_H_10_ClN_3_)_2_]Cl_2_·2H_2_O
*M* *_r_* = 743.42
Orthorhombic, 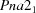

*a* = 10.1367 (5) Å
*b* = 16.2964 (7) Å
*c* = 17.8995 (8) Å
*V* = 2956.8 (2) Å^3^
*Z* = 4
Mo *K*α radiation
μ = 0.93 mm^−1^
*T* = 291 K
0.18 × 0.16 × 0.14 mm

#### Data collection

Bruker SMART CCD area-detector diffractometer
Absorption correction: multi-scan (*SADABS*; Bruker, 2000 ▶) *T*_min_ = 0.850, *T*_max_ = 0.881
15880 measured reflections
5166 independent reflections
4736 reflections with *I* > 2σ(*I*)
*R*_int_ = 0.069

#### Refinement

*R*[*F* ^2^ > 2σ(*F* ^2^)] = 0.034
*wR*(*F* ^2^) = 0.084
*S* = 1.01
5166 reflections
389 parameters
1 restraint
H-atom parameters constrained
Δρ_max_ = 0.78 e Å^−3^
Δρ_min_ = −0.42 e Å^−3^
Absolute structure: Flack (1983 ▶), 2475 Friedel pairs
Flack parameter: 0.47 (3)

### 

Data collection: *SMART* (Bruker, 2000 ▶); cell refinement: *SAINT* (Bruker, 2000 ▶); data reduction: *SAINT*; program(s) used to solve structure: *SHELXS97* (Sheldrick, 2008 ▶); program(s) used to refine structure: *SHELXL97* (Sheldrick, 2008 ▶); molecular graphics: *SHELXTL* (Sheldrick, 2008 ▶); software used to prepare material for publication: *SHELXTL*.

## Supplementary Material

Crystal structure: contains datablock(s) global, I. DOI: 10.1107/S1600536812021459/rz2748sup1.cif

Structure factors: contains datablock(s) I. DOI: 10.1107/S1600536812021459/rz2748Isup2.hkl

Additional supplementary materials:  crystallographic information; 3D view; checkCIF report

## Figures and Tables

**Table 1:** Hydrogen-bond geometry (Å, °)

*D*—H⋯*A*	*D*—H	H⋯*A*	*D*⋯*A*	*D*—H⋯*A*
C4—H4⋯Cl4	0.93	2.68	3.590 (5)	169
C12—H12⋯O2^i^	0.93	2.57	3.391 (6)	148
C15—H15⋯O1^ii^	0.93	2.48	3.179 (7)	132
C16—H16⋯Cl4^iii^	0.93	2.81	3.498 (5)	132
C27—H27⋯O1^iv^	0.93	2.60	3.474 (7)	158
C28—H28⋯Cl4^iv^	0.93	2.82	3.686 (4)	156
C30—H30⋯O2^ii^	0.93	2.54	3.203 (6)	128
O1—H1*A*⋯Cl4	0.85	2.76	3.231 (5)	116
O1—H1*B*⋯Cl4^v^	0.85	2.68	3.176 (4)	118
O2—H2*A*⋯Cl3	0.85	2.51	3.156 (4)	133
O2—H2*B*⋯Cl3^vi^	0.85	2.58	3.209 (4)	132

Symmetry codes: (i) 
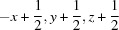
; (ii) 
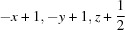
; (iii) 
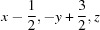
; (iv) 
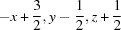
; (v) 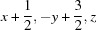; (vi) 
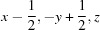
.

**Fig. 1.**
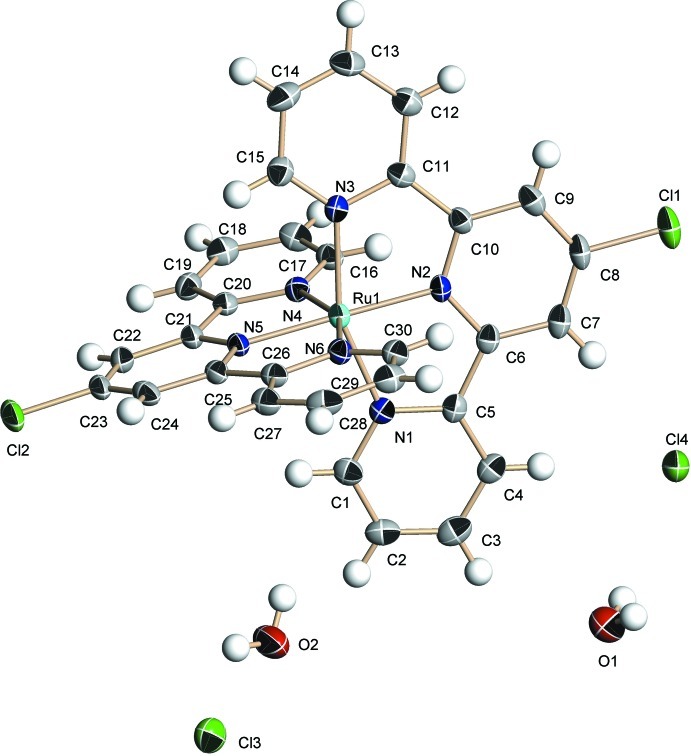
The asymmetric unit of the title compound with displacement ellipsoids drawn at the 30% probability level.

## supplementary crystallographic information

### Comment

The crystal structures of the hydrochlorate tetrafluoroborate and hydrochlorate hexafluorophosphorate derivatives (Huang & Qian, 2007*a*) and of the Ru(II), Cu(II), Zn(II), Ni(II), Fe(II), Cu(II) and Cd(II) complexes (Huang & Qian, 2007*b*; Beves *et al.*, 2008; You *et al.*, 2008; You *et al.*, 2009; Huang *et al.*, 2009) of 4'-chloro-2,2':6',2''-terpyridine with metal/ligand ratios of 1:1 and 1:2 have been recently reported by our group. As a continuation of the research in this field, we report herein the crystal structure of a ruthenium(II) dichloride complex bearing the same 4'-chloro-2,2':6',2''-terpyridine ligand with a 1:2 metal/ligand ratio.

The asymmetric unit of the title compound consists of a dication of formula [Ru(C_15_H_10_N_3_Cl)_2_]^2+^, two chloride anions and two water molecules (Fig. 1). In the cation, the ruthenium(II) metal displays a distorted octahedral geometry where each 4'-chloro-2,2':6',2'-terpyridine molecule acts as a tridentate *mer*-arranged N-ligand. The six Ru—N bond lengths (1.977 (3)—2.079 (4) Å) fall in the normal ranges of values. The two 4'-chloro-2,2':6',2''-terpyridine ligands are approximately planar (maximum deviation 0.156 (5) Å for atom C13) and the dihedral angle between them is 87.0 (3)°. In the crystal structure, cations, anions and water molecules are linked into a three-dimensional network by C—H···Cl, C—H···O and O—H···Cl hydrogen bonds (Table 1).

### Experimental

The title compound was obtained by refluxing *cis*-[RuCl_2_(DMSO)_4_] (0.121 g, 0.25 mmol) and 4'-chloro-2,2':6',2''-terpyridine (0.134 g, 0.50 mmol) in ethanol for 4 h [0.146 g; yield 78.4% based on Ru(II)]. Single crystals suitable for X-ray diffraction measurement were obtained after 10 days by slow evaporation of an ethanol/water solution (3:1 *v*/*v*) at room temperature in air. Elemental analysis: calculated for C_24_H_22_RuN_6_B_2_F_8_: C 43.08, H 3.31, N 12.56%; found: C 43.29, H 3.62, N 12.34%. Main FT–IR absorptions (KBr plates, cm^-1^): 3423 (b, s), 1630 (*s*), 1591 (w), 1421 (*m*), 1385 (*m*), 1107 (*m*), 1026 (w) and 793 (*m*).

### Refinement

The H atoms were placed in geometrically idealized positions (C—H = 0.93 Å and O—H = 0.85 Å) and refined as riding atoms, with *U*_iso_(H) = 1.2 *U*_eq_(C) or 1.5 *U*_eq_(O). The reported Flack parameter was obtained by TWIN/BASF procedure in SHELXL-97 (Sheldrick, 2008)

### Crystal data

**Table:**

[Ru(C_15_H_10_ClN_3_)_2_]Cl_2_·2H_2_O	*F*(000) = 1496
*M**_r_* = 743.42	*D*_x_ = 1.670 Mg m^−^^3^
Orthorhombic, *P**n**a*2_1_	Mo *K*α radiation, λ = 0.71073 Å
Hall symbol: P 2c -2n	Cell parameters from 8095 reflections
*a* = 10.1367 (5) Å	θ = 2.3–27.6°
*b* = 16.2964 (7) Å	µ = 0.93 mm^−^^1^
*c* = 17.8995 (8) Å	*T* = 291 K
*V* = 2956.8 (2) Å^3^	Block, red
*Z* = 4	0.18 × 0.16 × 0.14 mm

### Data collection

**Table:**

Bruker SMART CCD area-detector diffractometer	5166 independent reflections
Radiation source: fine-focus sealed tube	4736 reflections with *I* > 2σ(*I*)
Graphite monochromator	*R*_int_ = 0.069
φ and ω scans	θ_max_ = 25.0°, θ_min_ = 1.7°
Absorption correction: multi-scan (*SADABS*; Bruker, 2000)	*h* = −7→12
*T*_min_ = 0.850, *T*_max_ = 0.881	*k* = −19→19
15880 measured reflections	*l* = −21→20

### Refinement

**Table:**

Refinement on *F*^2^	Secondary atom site location: difference Fourier map
Least-squares matrix: full	Hydrogen site location: inferred from neighbouring sites
*R*[*F*^2^ > 2σ(*F*^2^)] = 0.034	H-atom parameters constrained
*wR*(*F*^2^) = 0.084	*w* = 1/[σ^2^(*F*_o_^2^) + (0.0507*P*)^2^] where *P* = (*F*_o_^2^ + 2*F*_c_^2^)/3
*S* = 1.01	(Δ/σ)_max_ = 0.001
5166 reflections	Δρ_max_ = 0.78 e Å^−^^3^
389 parameters	Δρ_min_ = −0.42 e Å^−^^3^
1 restraint	Absolute structure: Flack (1983), 2475 Friedel pairs
Primary atom site location: structure-invariant direct methods	Flack parameter: 0.47 (3)

### Special details

**Table:**

Experimental. The structure was solved by direct methods and successive difference Fourier syntheses.
Geometry. All e.s.d.'s (except the e.s.d. in the dihedral angle between two l.s. planes) are estimated using the full covariance matrix. The cell e.s.d.'s are taken into account individually in the estimation of e.s.d.'s in distances, angles and torsion angles; correlations between e.s.d.'s in cell parameters are only used when they are defined by crystal symmetry. An approximate (isotropic) treatment of cell e.s.d.'s is used for estimating e.s.d.'s involving l.s. planes.
Refinement. Refinement of *F*^2^ against ALL reflections. The weighted *R*-factor *wR* and goodness of fit *S* are based on *F*^2^, conventional *R*-factors *R* are based on *F*, with *F* set to zero for negative *F*^2^. The threshold expression of *F*^2^ > σ(*F*^2^) is used only for calculating *R*-factors(gt) *etc*. and is not relevant to the choice of reflections for refinement. *R*-factors based on *F*^2^ are statistically about twice as large as those based on *F*, and *R*- factors based on ALL data will be even larger.

### Fractional atomic coordinates and isotropic or equivalent isotropic displacement parameters (Å^2^)

**Table:**

	*x*	*y*	*z*	*U*_iso_*/*U*_eq_	
Ru1	0.29385 (2)	0.487615 (16)	0.79324 (2)	0.03652 (10)	
Cl1	0.31009 (13)	0.88127 (6)	0.76928 (7)	0.0702 (4)	
Cl2	0.29576 (11)	0.09346 (6)	0.80920 (7)	0.0620 (4)	
N1	0.4328 (4)	0.5037 (2)	0.7093 (2)	0.0403 (8)	
N2	0.2981 (3)	0.60884 (17)	0.7856 (3)	0.0387 (8)	
N3	0.1519 (3)	0.52045 (18)	0.87155 (19)	0.0405 (7)	
N4	0.1469 (3)	0.4589 (2)	0.71801 (18)	0.0383 (7)	
N5	0.2922 (3)	0.36654 (16)	0.8003 (3)	0.0366 (7)	
N6	0.4377 (4)	0.46780 (19)	0.8739 (2)	0.0402 (8)	
C1	0.4956 (4)	0.4461 (3)	0.6702 (2)	0.0495 (10)	
H1	0.4762	0.3914	0.6798	0.059*	
C2	0.5882 (5)	0.4642 (3)	0.6161 (3)	0.0555 (12)	
H2	0.6295	0.4222	0.5898	0.067*	
C3	0.6187 (4)	0.5436 (3)	0.6016 (3)	0.0590 (12)	
H3	0.6823	0.5565	0.5660	0.071*	
C4	0.5543 (4)	0.6052 (3)	0.6402 (2)	0.0497 (10)	
H4	0.5731	0.6599	0.6303	0.060*	
C5	0.4605 (4)	0.5844 (2)	0.6942 (2)	0.0419 (9)	
C6	0.3842 (4)	0.6443 (2)	0.7371 (2)	0.0415 (9)	
C7	0.3893 (4)	0.7289 (2)	0.7306 (2)	0.0490 (10)	
H7	0.4468	0.7541	0.6972	0.059*	
C8	0.3062 (4)	0.7747 (2)	0.7753 (2)	0.0487 (13)	
C9	0.2190 (4)	0.7392 (3)	0.8253 (3)	0.0488 (10)	
H9	0.1661	0.7703	0.8570	0.059*	
C10	0.2161 (4)	0.6537 (2)	0.8288 (2)	0.0389 (9)	
C11	0.1303 (4)	0.6035 (2)	0.8758 (2)	0.0415 (9)	
C12	0.0307 (4)	0.6351 (3)	0.9204 (2)	0.0513 (10)	
H12	0.0168	0.6914	0.9229	0.062*	
C13	−0.0476 (4)	0.5823 (3)	0.9609 (3)	0.0584 (12)	
H13	−0.1157	0.6027	0.9904	0.070*	
C14	−0.0248 (6)	0.5004 (4)	0.9576 (4)	0.0618 (15)	
H14	−0.0772	0.4641	0.9846	0.074*	
C15	0.0791 (5)	0.4711 (3)	0.9129 (3)	0.0494 (11)	
H15	0.0971	0.4151	0.9125	0.059*	
C16	0.0690 (5)	0.5099 (3)	0.6799 (3)	0.0460 (11)	
H16	0.0848	0.5660	0.6838	0.055*	
C17	−0.0323 (6)	0.4844 (3)	0.6356 (3)	0.0523 (13)	
H17	−0.0843	0.5226	0.6107	0.063*	
C18	−0.0577 (4)	0.4009 (3)	0.6278 (2)	0.0534 (11)	
H18	−0.1270	0.3822	0.5984	0.064*	
C19	0.0235 (4)	0.3467 (3)	0.6653 (2)	0.0468 (10)	
H19	0.0115	0.2904	0.6599	0.056*	
C20	0.1226 (4)	0.3762 (2)	0.7108 (2)	0.0400 (9)	
C21	0.2080 (4)	0.3234 (2)	0.7571 (2)	0.0381 (8)	
C22	0.2096 (4)	0.2384 (2)	0.7582 (2)	0.0451 (10)	
H22	0.1552	0.2083	0.7266	0.054*	
C23	0.2942 (4)	0.1997 (2)	0.8073 (3)	0.0443 (11)	
C24	0.3774 (4)	0.2437 (2)	0.8535 (2)	0.0467 (10)	
H24	0.4327	0.2170	0.8870	0.056*	
C25	0.3762 (4)	0.3286 (2)	0.8485 (2)	0.0384 (9)	
C26	0.4577 (4)	0.3863 (2)	0.8907 (2)	0.0410 (9)	
C27	0.5518 (4)	0.3629 (3)	0.9433 (3)	0.0501 (10)	
H27	0.5654	0.3077	0.9541	0.060*	
C28	0.6242 (4)	0.4223 (3)	0.9789 (2)	0.0559 (12)	
H28	0.6876	0.4072	1.0139	0.067*	
C29	0.6039 (5)	0.5037 (3)	0.9635 (3)	0.0548 (13)	
H29	0.6520	0.5441	0.9880	0.066*	
C30	0.5092 (4)	0.5244 (3)	0.9101 (2)	0.0472 (10)	
H30	0.4953	0.5795	0.8992	0.057*	
O1	0.8180 (4)	0.6796 (3)	0.5095 (3)	0.0911 (12)	
H1A	0.7593	0.7075	0.4870	0.137*	
H1B	0.8513	0.7082	0.5445	0.137*	
O2	0.6110 (4)	0.3114 (2)	0.4811 (2)	0.0952 (14)	
H2A	0.6240	0.2689	0.5077	0.143*	
H2B	0.5485	0.3394	0.4998	0.143*	
Cl3	0.82738 (13)	0.20222 (8)	0.55975 (9)	0.0755 (4)	
Cl4	0.60880 (13)	0.81073 (7)	0.57497 (8)	0.0725 (4)	

### Atomic displacement parameters (Å^2^)

**Table:**

	*U*^11^	*U*^22^	*U*^33^	*U*^12^	*U*^13^	*U*^23^
Ru1	0.04084 (15)	0.02974 (15)	0.03897 (15)	0.00017 (10)	−0.00122 (19)	0.00334 (18)
Cl1	0.0967 (8)	0.0306 (5)	0.0833 (11)	−0.0047 (5)	−0.0244 (7)	0.0081 (5)
Cl2	0.0896 (8)	0.0288 (4)	0.0676 (10)	0.0044 (5)	0.0194 (6)	0.0067 (5)
N1	0.040 (2)	0.0425 (19)	0.039 (2)	−0.0004 (15)	−0.0031 (16)	0.0033 (15)
N2	0.0425 (15)	0.0282 (14)	0.045 (2)	−0.0023 (11)	−0.0037 (16)	0.0026 (18)
N3	0.0423 (18)	0.0379 (19)	0.0412 (19)	−0.0049 (14)	−0.0018 (15)	0.0015 (14)
N4	0.0407 (17)	0.0389 (17)	0.0354 (18)	−0.0009 (14)	0.0011 (14)	0.0015 (14)
N5	0.0437 (15)	0.0285 (13)	0.0377 (19)	0.0032 (11)	0.0077 (16)	0.0027 (18)
N6	0.044 (2)	0.0368 (16)	0.040 (2)	0.0002 (16)	0.0019 (15)	0.0035 (16)
C1	0.049 (2)	0.051 (3)	0.049 (3)	0.0090 (19)	−0.004 (2)	0.003 (2)
C2	0.045 (3)	0.074 (3)	0.047 (3)	0.009 (3)	0.001 (2)	0.000 (3)
C3	0.044 (3)	0.082 (4)	0.050 (3)	−0.003 (2)	−0.004 (2)	0.008 (3)
C4	0.047 (2)	0.058 (3)	0.043 (2)	−0.0064 (19)	−0.0035 (19)	0.0075 (19)
C5	0.040 (2)	0.041 (2)	0.045 (2)	−0.0038 (16)	−0.0098 (18)	0.0067 (17)
C6	0.043 (2)	0.039 (2)	0.043 (2)	−0.0068 (17)	−0.0092 (18)	0.0042 (16)
C7	0.058 (3)	0.039 (2)	0.049 (3)	−0.0110 (19)	−0.013 (2)	0.0118 (18)
C8	0.064 (3)	0.0271 (18)	0.055 (4)	−0.0050 (16)	−0.020 (2)	0.0069 (17)
C9	0.057 (3)	0.037 (2)	0.052 (2)	0.0085 (18)	−0.015 (2)	−0.0036 (18)
C10	0.041 (2)	0.034 (2)	0.042 (2)	0.0035 (15)	−0.0067 (17)	−0.0009 (16)
C11	0.039 (2)	0.047 (2)	0.039 (2)	0.0063 (16)	−0.0065 (17)	−0.0016 (17)
C12	0.054 (3)	0.054 (3)	0.045 (3)	0.005 (2)	−0.004 (2)	−0.0063 (19)
C13	0.052 (3)	0.073 (3)	0.050 (3)	0.008 (2)	0.004 (2)	−0.017 (2)
C14	0.053 (4)	0.082 (4)	0.050 (3)	−0.013 (3)	0.009 (3)	−0.002 (2)
C15	0.057 (3)	0.047 (2)	0.044 (3)	−0.007 (2)	0.000 (2)	−0.002 (2)
C16	0.049 (3)	0.043 (2)	0.046 (3)	0.0086 (19)	0.002 (2)	0.0062 (19)
C17	0.057 (3)	0.060 (3)	0.041 (3)	0.013 (2)	−0.007 (2)	0.002 (2)
C18	0.047 (3)	0.069 (3)	0.044 (3)	−0.004 (2)	−0.004 (2)	−0.006 (2)
C19	0.046 (2)	0.049 (2)	0.046 (2)	−0.0081 (18)	0.0012 (19)	−0.0030 (18)
C20	0.043 (2)	0.039 (2)	0.039 (2)	0.0009 (16)	0.0083 (17)	−0.0016 (16)
C21	0.042 (2)	0.032 (2)	0.041 (2)	0.0008 (15)	0.0046 (16)	0.0014 (16)
C22	0.049 (2)	0.035 (2)	0.051 (2)	−0.0050 (16)	0.0085 (18)	−0.0046 (18)
C23	0.059 (2)	0.0281 (17)	0.046 (3)	0.0025 (16)	0.0182 (19)	0.0032 (17)
C24	0.056 (2)	0.041 (2)	0.043 (2)	0.0148 (18)	0.0087 (19)	0.0076 (17)
C25	0.045 (2)	0.0344 (19)	0.035 (2)	0.0035 (16)	0.0074 (17)	0.0060 (15)
C26	0.042 (2)	0.044 (2)	0.037 (2)	0.0072 (17)	0.0027 (17)	0.0048 (16)
C27	0.054 (3)	0.051 (3)	0.045 (2)	0.010 (2)	0.0010 (19)	0.0077 (19)
C28	0.044 (3)	0.081 (3)	0.043 (3)	0.011 (2)	−0.0042 (19)	0.002 (2)
C29	0.049 (3)	0.068 (3)	0.047 (3)	−0.004 (2)	−0.002 (2)	−0.008 (2)
C30	0.045 (2)	0.046 (3)	0.051 (3)	−0.0013 (19)	0.001 (2)	−0.003 (2)
O1	0.094 (3)	0.079 (3)	0.100 (3)	0.001 (2)	0.011 (2)	−0.014 (2)
O2	0.109 (3)	0.067 (2)	0.109 (3)	0.024 (2)	0.035 (3)	0.012 (2)
Cl3	0.0685 (7)	0.0689 (8)	0.0892 (10)	−0.0103 (6)	−0.0019 (7)	−0.0065 (7)
Cl4	0.0767 (8)	0.0468 (6)	0.0941 (10)	−0.0041 (5)	0.0233 (7)	0.0034 (6)

### Geometric parameters (Å, º)

**Table:**

Ru1—N1	2.075 (4)	C11—C12	1.387 (6)
Ru1—N2	1.981 (3)	C12—C13	1.377 (7)
Ru1—N3	2.079 (4)	C12—H12	0.9300
Ru1—N4	2.062 (3)	C13—C14	1.355 (7)
Ru1—N5	1.977 (3)	C13—H13	0.9300
Ru1—N6	2.077 (4)	C14—C15	1.407 (8)
Cl1—C8	1.740 (4)	C14—H14	0.9300
Cl2—C23	1.732 (4)	C15—H15	0.9300
N1—C1	1.333 (6)	C16—C17	1.362 (7)
N1—C5	1.371 (5)	C16—H16	0.9300
N2—C10	1.350 (5)	C17—C18	1.392 (6)
N2—C6	1.360 (5)	C17—H17	0.9300
N3—C15	1.318 (6)	C18—C19	1.381 (6)
N3—C11	1.373 (5)	C18—H18	0.9300
N4—C16	1.334 (5)	C19—C20	1.379 (5)
N4—C20	1.376 (5)	C19—H19	0.9300
N5—C21	1.348 (5)	C20—C21	1.475 (5)
N5—C25	1.361 (5)	C21—C22	1.385 (6)
N6—C30	1.340 (5)	C22—C23	1.380 (6)
N6—C26	1.377 (5)	C22—H22	0.9300
C1—C2	1.380 (7)	C23—C24	1.382 (6)
C1—H1	0.9300	C24—C25	1.387 (5)
C2—C3	1.355 (7)	C24—H24	0.9300
C2—H2	0.9300	C25—C26	1.462 (6)
C3—C4	1.383 (7)	C26—C27	1.393 (6)
C3—H3	0.9300	C27—C28	1.373 (7)
C4—C5	1.398 (6)	C27—H27	0.9300
C4—H4	0.9300	C28—C29	1.370 (7)
C5—C6	1.463 (5)	C28—H28	0.9300
C6—C7	1.385 (6)	C29—C30	1.396 (7)
C7—C8	1.380 (6)	C29—H29	0.9300
C7—H7	0.9300	C30—H30	0.9300
C8—C9	1.386 (6)	O1—H1A	0.8501
C9—C10	1.394 (6)	O1—H1B	0.8500
C9—H9	0.9299	O2—H2A	0.8500
C10—C11	1.462 (6)	O2—H2B	0.8500
			
N5—Ru1—N2	179.19 (14)	N3—C11—C12	120.9 (4)
N5—Ru1—N4	78.99 (14)	N3—C11—C10	115.2 (3)
N2—Ru1—N4	101.35 (14)	C12—C11—C10	123.8 (4)
N5—Ru1—N1	100.25 (14)	C13—C12—C11	119.4 (4)
N2—Ru1—N1	79.01 (15)	C13—C12—H12	120.3
N4—Ru1—N1	92.66 (14)	C11—C12—H12	120.3
N5—Ru1—N6	78.84 (14)	C14—C13—C12	119.6 (5)
N2—Ru1—N6	100.83 (14)	C14—C13—H13	120.2
N4—Ru1—N6	157.79 (12)	C12—C13—H13	120.2
N1—Ru1—N6	92.64 (11)	C13—C14—C15	119.2 (5)
N5—Ru1—N3	102.01 (14)	C13—C14—H14	120.4
N2—Ru1—N3	78.74 (14)	C15—C14—H14	120.4
N4—Ru1—N3	89.93 (12)	N3—C15—C14	122.1 (5)
N1—Ru1—N3	157.68 (12)	N3—C15—H15	119.0
N6—Ru1—N3	93.28 (14)	C14—C15—H15	119.0
C1—N1—C5	118.3 (4)	N4—C16—C17	123.6 (4)
C1—N1—Ru1	128.0 (3)	N4—C16—H16	118.2
C5—N1—Ru1	113.8 (3)	C17—C16—H16	118.2
C10—N2—C6	122.0 (3)	C16—C17—C18	119.7 (4)
C10—N2—Ru1	119.2 (3)	C16—C17—H17	120.1
C6—N2—Ru1	118.8 (3)	C18—C17—H17	120.1
C15—N3—C11	118.7 (4)	C19—C18—C17	117.8 (4)
C15—N3—Ru1	127.5 (3)	C19—C18—H18	121.1
C11—N3—Ru1	113.7 (3)	C17—C18—H18	121.1
C16—N4—C20	117.2 (4)	C20—C19—C18	119.8 (4)
C16—N4—Ru1	128.3 (3)	C20—C19—H19	120.1
C20—N4—Ru1	114.4 (3)	C18—C19—H19	120.1
C21—N5—C25	121.5 (3)	N4—C20—C19	121.8 (4)
C21—N5—Ru1	119.3 (3)	N4—C20—C21	114.4 (3)
C25—N5—Ru1	119.2 (3)	C19—C20—C21	123.8 (3)
C30—N6—C26	118.5 (4)	N5—C21—C22	120.4 (4)
C30—N6—Ru1	127.5 (3)	N5—C21—C20	112.9 (3)
C26—N6—Ru1	113.9 (3)	C22—C21—C20	126.7 (4)
N1—C1—C2	122.9 (4)	C23—C22—C21	118.2 (4)
N1—C1—H1	118.5	C23—C22—H22	120.9
C2—C1—H1	118.5	C21—C22—H22	120.9
C3—C2—C1	119.6 (5)	C22—C23—C24	121.6 (4)
C3—C2—H2	120.2	C22—C23—Cl2	118.4 (3)
C1—C2—H2	120.2	C24—C23—Cl2	120.0 (3)
C2—C3—C4	119.3 (4)	C23—C24—C25	118.3 (4)
C2—C3—H3	120.4	C23—C24—H24	120.9
C4—C3—H3	120.4	C25—C24—H24	120.9
C3—C4—C5	119.4 (4)	N5—C25—C24	119.9 (4)
C3—C4—H4	120.3	N5—C25—C26	112.9 (3)
C5—C4—H4	120.3	C24—C25—C26	127.1 (3)
N1—C5—C4	120.5 (4)	N6—C26—C27	120.9 (4)
N1—C5—C6	115.4 (4)	N6—C26—C25	115.1 (3)
C4—C5—C6	124.1 (4)	C27—C26—C25	124.0 (4)
N2—C6—C7	120.0 (4)	C28—C27—C26	119.1 (4)
N2—C6—C5	113.0 (3)	C28—C27—H27	120.5
C7—C6—C5	127.0 (4)	C26—C27—H27	120.5
C8—C7—C6	117.9 (4)	C29—C28—C27	120.6 (4)
C8—C7—H7	121.1	C29—C28—H28	119.7
C6—C7—H7	121.1	C27—C28—H28	119.7
C7—C8—C9	122.5 (4)	C28—C29—C30	118.4 (5)
C7—C8—Cl1	119.4 (3)	C28—C29—H29	120.8
C9—C8—Cl1	118.1 (3)	C30—C29—H29	120.8
C8—C9—C10	117.3 (4)	N6—C30—C29	122.5 (4)
C8—C9—H9	122.3	N6—C30—H30	118.8
C10—C9—H9	120.4	C29—C30—H30	118.8
N2—C10—C9	120.2 (4)	H1A—O1—H1B	109.5
N2—C10—C11	113.1 (3)	H2A—O2—H2B	109.5
C9—C10—C11	126.7 (4)		
			
N5—Ru1—N1—C1	3.0 (4)	C4—C5—C6—C7	1.6 (6)
N2—Ru1—N1—C1	−177.4 (4)	N2—C6—C7—C8	0.9 (5)
N4—Ru1—N1—C1	−76.3 (4)	C5—C6—C7—C8	178.4 (4)
N6—Ru1—N1—C1	82.1 (4)	C6—C7—C8—C9	−0.3 (6)
N3—Ru1—N1—C1	−172.6 (3)	C6—C7—C8—Cl1	179.6 (3)
N5—Ru1—N1—C5	−176.9 (3)	C7—C8—C9—C10	−0.8 (6)
N2—Ru1—N1—C5	2.7 (3)	Cl1—C8—C9—C10	179.3 (3)
N4—Ru1—N1—C5	103.8 (3)	C6—N2—C10—C9	−0.8 (6)
N6—Ru1—N1—C5	−97.8 (3)	Ru1—N2—C10—C9	178.8 (3)
N3—Ru1—N1—C5	7.5 (6)	C6—N2—C10—C11	179.0 (3)
N4—Ru1—N2—C10	87.1 (3)	Ru1—N2—C10—C11	−1.3 (5)
N1—Ru1—N2—C10	177.6 (3)	C8—C9—C10—N2	1.4 (5)
N6—Ru1—N2—C10	−91.8 (3)	C8—C9—C10—C11	−178.4 (4)
N3—Ru1—N2—C10	−0.6 (3)	C15—N3—C11—C12	−2.5 (6)
N4—Ru1—N2—C6	−93.2 (3)	Ru1—N3—C11—C12	174.2 (3)
N1—Ru1—N2—C6	−2.7 (3)	C15—N3—C11—C10	179.4 (4)
N6—Ru1—N2—C6	87.9 (3)	Ru1—N3—C11—C10	−3.9 (4)
N3—Ru1—N2—C6	179.1 (3)	N2—C10—C11—N3	3.5 (5)
N5—Ru1—N3—C15	−1.5 (4)	C9—C10—C11—N3	−176.7 (4)
N2—Ru1—N3—C15	178.8 (4)	N2—C10—C11—C12	−174.6 (4)
N4—Ru1—N3—C15	77.2 (4)	C9—C10—C11—C12	5.2 (6)
N1—Ru1—N3—C15	174.1 (4)	N3—C11—C12—C13	0.0 (6)
N6—Ru1—N3—C15	−80.8 (4)	C10—C11—C12—C13	178.0 (4)
N5—Ru1—N3—C11	−177.8 (3)	C11—C12—C13—C14	1.1 (7)
N2—Ru1—N3—C11	2.5 (3)	C12—C13—C14—C15	0.2 (8)
N4—Ru1—N3—C11	−99.1 (3)	C11—N3—C15—C14	3.9 (7)
N1—Ru1—N3—C11	−2.3 (5)	Ru1—N3—C15—C14	−172.3 (4)
N6—Ru1—N3—C11	102.9 (3)	C13—C14—C15—N3	−2.8 (8)
N5—Ru1—N4—C16	175.6 (4)	C20—N4—C16—C17	0.6 (6)
N2—Ru1—N4—C16	−5.1 (4)	Ru1—N4—C16—C17	−174.8 (4)
N1—Ru1—N4—C16	−84.4 (4)	N4—C16—C17—C18	−0.8 (8)
N6—Ru1—N4—C16	171.9 (4)	C16—C17—C18—C19	−0.8 (7)
N3—Ru1—N4—C16	73.4 (4)	C17—C18—C19—C20	2.4 (6)
N5—Ru1—N4—C20	0.1 (3)	C16—N4—C20—C19	1.1 (5)
N2—Ru1—N4—C20	179.4 (3)	Ru1—N4—C20—C19	177.1 (3)
N1—Ru1—N4—C20	100.0 (3)	C16—N4—C20—C21	−177.3 (3)
N6—Ru1—N4—C20	−3.6 (5)	Ru1—N4—C20—C21	−1.2 (4)
N3—Ru1—N4—C20	−102.1 (3)	C18—C19—C20—N4	−2.6 (6)
N4—Ru1—N5—C21	1.1 (3)	C18—C19—C20—C21	175.5 (4)
N1—Ru1—N5—C21	−89.6 (3)	C25—N5—C21—C22	−3.3 (6)
N6—Ru1—N5—C21	179.7 (3)	Ru1—N5—C21—C22	176.7 (3)
N3—Ru1—N5—C21	88.7 (3)	C25—N5—C21—C20	178.0 (3)
N4—Ru1—N5—C25	−178.9 (3)	Ru1—N5—C21—C20	−2.1 (5)
N1—Ru1—N5—C25	90.3 (3)	N4—C20—C21—N5	2.1 (5)
N6—Ru1—N5—C25	−0.3 (3)	C19—C20—C21—N5	−176.2 (4)
N3—Ru1—N5—C25	−91.4 (3)	N4—C20—C21—C22	−176.6 (4)
N5—Ru1—N6—C30	−179.7 (4)	C19—C20—C21—C22	5.1 (6)
N2—Ru1—N6—C30	1.1 (4)	N5—C21—C22—C23	3.5 (6)
N4—Ru1—N6—C30	−176.0 (3)	C20—C21—C22—C23	−178.0 (4)
N1—Ru1—N6—C30	80.4 (4)	C21—C22—C23—C24	−1.2 (6)
N3—Ru1—N6—C30	−78.1 (3)	C21—C22—C23—Cl2	179.6 (3)
N5—Ru1—N6—C26	−0.3 (3)	C22—C23—C24—C25	−1.3 (6)
N2—Ru1—N6—C26	−179.5 (3)	Cl2—C23—C24—C25	177.9 (3)
N4—Ru1—N6—C26	3.4 (6)	C21—N5—C25—C24	0.7 (6)
N1—Ru1—N6—C26	−100.2 (3)	Ru1—N5—C25—C24	−179.2 (3)
N3—Ru1—N6—C26	101.3 (3)	C21—N5—C25—C26	−179.2 (3)
C5—N1—C1—C2	0.9 (6)	Ru1—N5—C25—C26	0.8 (4)
Ru1—N1—C1—C2	−179.0 (3)	C23—C24—C25—N5	1.5 (5)
N1—C1—C2—C3	0.4 (7)	C23—C24—C25—C26	−178.6 (4)
C1—C2—C3—C4	−1.3 (6)	C30—N6—C26—C27	−1.1 (6)
C2—C3—C4—C5	0.9 (6)	Ru1—N6—C26—C27	179.4 (3)
C1—N1—C5—C4	−1.3 (6)	C30—N6—C26—C25	−179.7 (3)
Ru1—N1—C5—C4	178.6 (3)	Ru1—N6—C26—C25	0.8 (4)
C1—N1—C5—C6	177.6 (3)	N5—C25—C26—N6	−1.1 (5)
Ru1—N1—C5—C6	−2.4 (4)	C24—C25—C26—N6	179.0 (4)
C3—C4—C5—N1	0.4 (6)	N5—C25—C26—C27	−179.6 (4)
C3—C4—C5—C6	−178.5 (4)	C24—C25—C26—C27	0.5 (6)
C10—N2—C6—C7	−0.3 (6)	N6—C26—C27—C28	0.6 (6)
Ru1—N2—C6—C7	180.0 (3)	C25—C26—C27—C28	179.1 (4)
C10—N2—C6—C5	−178.2 (4)	C26—C27—C28—C29	0.4 (7)
Ru1—N2—C6—C5	2.2 (4)	C27—C28—C29—C30	−0.8 (7)
N1—C5—C6—N2	0.3 (5)	C26—N6—C30—C29	0.7 (6)
C4—C5—C6—N2	179.2 (4)	Ru1—N6—C30—C29	−179.9 (3)
N1—C5—C6—C7	−177.3 (4)	C28—C29—C30—N6	0.3 (7)

### Hydrogen-bond geometry (Å, º)

**Table:**

*D*—H···*A*	*D*—H	H···*A*	*D*···*A*	*D*—H···*A*
C4—H4···Cl4	0.93	2.68	3.590 (5)	169
C12—H12···O2^i^	0.93	2.57	3.391 (6)	148
C15—H15···O1^ii^	0.93	2.48	3.179 (7)	132
C16—H16···Cl4^iii^	0.93	2.81	3.498 (5)	132
C27—H27···O1^iv^	0.93	2.60	3.474 (7)	158
C28—H28···Cl4^iv^	0.93	2.82	3.686 (4)	156
C30—H30···O2^ii^	0.93	2.54	3.203 (6)	128
O1—H1*A*···Cl4	0.85	2.76	3.231 (5)	116
O1—H1*B*···Cl4^v^	0.85	2.68	3.176 (4)	118
O2—H2*A*···Cl3	0.85	2.51	3.156 (4)	133
O2—H2*B*···Cl3^vi^	0.85	2.58	3.209 (4)	132

Symmetry codes: (i) −*x*+1/2, *y*+1/2, *z*+1/2; (ii) −*x*+1, −*y*+1, *z*+1/2; (iii) *x*−1/2, −*y*+3/2, *z*; (iv) −*x*+3/2, *y*−1/2, *z*+1/2; (v) *x*+1/2, −*y*+3/2, *z*; (vi) *x*−1/2, −*y*+1/2, *z*.
